# Elevated pre-treatment levels of plasma C-reactive protein are associated with poor prognosis after breast cancer: a cohort study

**DOI:** 10.1186/bcr2891

**Published:** 2011-06-03

**Authors:** Kristine H Allin, Børge G Nordestgaard, Henrik Flyger, Stig E Bojesen

**Affiliations:** 1 Department of Clinical Biochemistry Herlev Hospital, Copenhagen University Hospital, Herlev Ringvej 75, Herlev, DK-2730, Denmark; 2 Faculty of Health Sciences, University of Copenhagen, Blegdamsvej 3, Copenhagen, DK-2200, Denmark; 3 Department of Breast Surgery Herlev Hospital, Copenhagen University Hospital, Herlev Ringvej 75, Herlev, DK-2730, Denmark

## Abstract

**Introduction:**

We examined whether plasma C-reactive protein (CRP) levels at the time of diagnosis of breast cancer are associated with overall survival, disease-free survival, death from breast cancer, and recurrence of breast cancer.

**Methods:**

We observed 2,910 women for up to seven years after they were diagnosed with invasive breast cancer (median follow-up time was three years). Plasma levels of high-sensitivity CRP were measured at the time of diagnosis and we assessed the association between CRP levels and risk of reduced overall and disease-free survival, death from breast cancer, and recurrence of breast cancer by using the Kaplan-Meier method and Cox proportional hazards regression. During follow-up, 383 women died (225 of whom died from breast cancer) and 118 women experienced recurrence of breast cancer.

**Results:**

Elevated CRP levels across tertiles at the time of diagnosis were associated with reduced overall and disease-free survival and with increased risk of death from breast cancer (log-rank trend for all, *P *< 0.001), but not with recurrence. The multifactor-adjusted hazard ratios (HR) of reduced overall survival among women in the middle and highest versus the lowest tertile of CRP were 1.30 (95% CI, 0.97 to 1.73) and 1.94 (1.48 to 2.55), respectively. Corresponding HRs of reduced disease-free survival were 1.16 (0.89 to 1.50) and 1.76 (1.38 to 2.25) and of death from breast cancer 1.22 (0.84 to 1.78) and 1.66 (1.15 to 2.41). Dividing CRP levels into octiles resulted in a stepwise increased risk of reduced overall survival (*P *for trend <0.001) and the multifactor-adjusted HR among women in the highest versus the lowest octile of CRP was 2.51 (1.53 to 4.12). Compared to women with CRP levels in the 0 to 25% percentile (<0.78 mg/L), the multifactor-adjusted HR of reduced overall survival among women with CRP levels ≥95% percentile (≥16.4 mg/L) was 3.58 (2.36 to 5.42). Among women with HER2-positive tumours, the multifactor-adjusted HR of reduced overall survival for the highest versus the lowest tertile of CRP was 8.63 (2.04 to 36.4).

**Conclusions:**

Elevated CRP levels at the time of diagnosis of breast cancer are associated with reduced overall and disease-free survival and with increased risk of death from breast cancer.

## Introduction

Elevated plasma levels of C-reactive protein (CRP) may be associated with poor prognosis after breast cancer. CRP is a classical acute-phase protein displaying rapid and pronounced rise of its plasma concentration in response to acute inflammation, infection, and tissue damage [1,2]. Circulating levels of CRP are also moderately elevated during chronic inflammatory diseases and cancer [3]. CRP is produced in the liver, predominantly under transcriptional control by the cytokine interleukin-6 originating from the site of pathology [4]. Many tumours arise at sites of chronic inflammation or they trigger inflammatory responses that result in the formation of an inflammatory microenvironment around the tumour [5-9]. In fact, it has recently been suggested that cancer-related inflammation may represent the seventh hallmark of cancer in addition to the six hallmarks identified by Hanahan and Weinberg [6]. Since inflammation in the tumour microenvironment stimulates tumour growth, invasion, and metastasis, inflammation seems to favour invasion and metastasis more than to mount an effective host anti-tumour response [5-9].

Previous epidemiologic studies have reported that elevated CRP levels may be associated with poor prognosis of several types of solid cancers [10], including endometrial [11], cervical [12], colorectal, pancreatic, hepatocellular, esophageal, renal cell, bladder, prostate, ovarian, and non-small-cell lung cancer [13,14]. Although breast cancers rarely are characterized by significant histological inflammation, emerging evidence nevertheless suggests that inflammatory pathways also play an important role in breast cancer progression [15-20]. However, results from prospective epidemiologic studies are conflicting, with some studies showing an association between elevated CRP levels and poor prognosis [21-24] and others showing no association [25,26]. The largest study so far comprised 700 women treated successfully for early stage breast cancer and found that elevated levels of CRP measured two and a half years after the time of diagnosis were associated with reduced disease-free and overall survival [23]. Thus, it is unclear whether CRP levels measured at the time of diagnosis are associated with breast cancer prognosis.

We used a prospective cohort study of 2,910 patients with invasive breast cancer to examine whether plasma CRP levels at the time of diagnosis of breast cancer are associated with overall survival, disease-free survival, death from breast cancer, and recurrence of breast cancer.

## Materials and methods

### Patients

We studied patients with invasive breast cancer from the Copenhagen Breast Cancer Study, which is an ongoing cohort study of Danish breast cancer patients. Since January 2002, all patients that are referred to the Department of Breast Surgery, Herlev Hospital, Copenhagen University Hospital with suspected breast cancer are asked to participate in the study (participation rate 93%). At study enrolment the patients answer a questionnaire regarding sociodemographic factors, lifestyle factors, anthropometric characteristics, medical history, and family history of cancer. Furthermore, they give blood for measurements of biochemical parameters.

**Figure 1:**
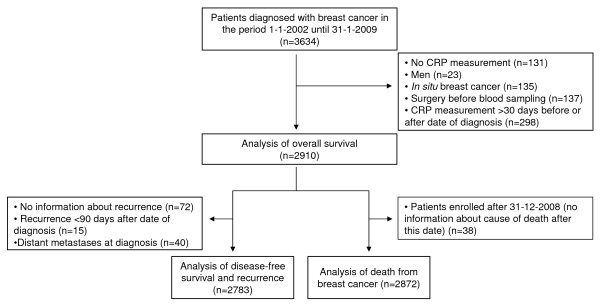
**Flowchart illustrating enrolment and exclusions**. CRP, C-reactive protein.

In the present study, we included patients who were diagnosed with breast cancer in the period 1 January 2002 until 31 January 2009 (n = 3,634) (Figure 1). Since our intention was to study the association between CRP levels and prognosis among women with invasive breast cancer, we excluded patients without a measurement of CRP (n = 131), men (n = 23), and women with *in situ *breast cancer (n = 135). To avoid potential bias by the influence of surgery on CRP levels, we also excluded women who underwent breast cancer surgery before blood sampling (n = 137). Finally, to ensure that plasma CRP was measured at the time of diagnosis, we excluded women who had plasma CRP measured more than 30 days before or after the date of diagnosis of breast cancer (n = 298). Thus, we included 2,910 women aged 26 to 99 years in the analysis of overall survival. For the analysis of disease-free survival and recurrence, we additionally excluded 72 women for whom information about recurrence was missing, 15 individuals who, according to the register, had recurrence less than 90 days after their date of diagnosis, and 40 women who had distant metastasis at diagnosis. The latter two groups were excluded to ensure that the registered recurrences were indeed true recurrences. Thus, 2,783 women aged 26 to 99 years were included in the analysis of disease-free survival and recurrence. For the analysis of death from breast cancer, we excluded 38 women who were enrolled after 31 December 2008, since we did not have information about cause-specific death after this date. Thus, we included 2,872 women aged 26 to 99 years in the analysis of death from breast cancer (Figure 1). Follow-up time for each patient began at blood sampling and ended for overall survival at occurrence of death, emigration, or May 2009, whichever came first. For disease-free survival and recurrence, follow-up time ended at occurrence of recurrence, death, emigration, or May 2009, whichever came first. For death from breast cancer, follow-up time ended at death from breast cancer, death from other causes, emigration, or December 2008, whichever came first. The median follow-up period was three years (range = 0 to 7 years). During follow-up, 383 women died, 225 women died from breast cancer, 118 women experienced recurrence of breast cancer, and 4 women emigrated.

### Register information

Information about diagnoses and prognostic tumour characteristics (tumour size, lymph node status, presence of distant metastases, tumour grade, and estrogen receptor, progesterone receptor, and HER2 status) of index breast cancers was obtained from the national Danish Breast Cancer Cooperative Group (DBCG) [27]. Diagnoses and prognostic tumour characteristics were pathologically verified by clinical pathologists. Date of recurrence was obtained from DBCG and date of death and emigration was obtained from the national Danish Civil Registration System, which is 100% complete. Information about death from breast cancer was obtained from the National Danish Causes of Death Registry in which all death certificates in Denmark are registered. Danish death certificates register courses of diseases which lead to the death, with the underlying cause of death being the disease which started this course. In the present study, patients who were registered with the World Health Organization (WHO) *International Classification of Diseases (ICD), Tenth Revision *(*ICD-10*) codes C50.0 to C50.9 as the underlying cause of death were recorded as having died from breast cancer.

Information about diagnosis of cardiovascular diseases was obtained from the national Danish Patient Registry which all public and private hospitals in Denmark report to. Diagnoses were classified according to the WHO *ICD, Eighth Revision *(*ICD-8 *codes 410 to 414 and 432 to 435) or *Tenth Revision *(*ICD-10 *codes I20 to I25 and I63 to I64 and G45).

### Ethics

The ethical committee of Copenhagen and Frederiksberg, Denmark approved the study (KA-02152). All patients gave written informed consent.

### CRP analysis

We determined the plasma CRP levels in fresh plasma samples by using a high-sensitivity nephelometry assay (CardioPhase hsCRP, code OQIY21, Dade Behring, Deerfield, IL, USA) performed on a Behring Nephelometer or a high-sensitivity turbidimetry assay (Code LX001, Dako, Glostrup, Denmark) performed on a Konelab 60 analyzer. All analyses were performed in the same laboratory at the Department of Clinical Biochemistry, Herlev Hospital, Copenhagen University Hospital. The detection limit was 0.17 mg/L for the nephelometry assay and 0.20 mg/L for the turbidimetry assay. Prior to the switch from the nephelometry to the turbidimetry assay, the two assays were compared by measuring plasma CRP with each method in 29 individuals. Spearman rank correlation coefficient between the two CRP assays was 0.96, and a Wilcoxon signed-rank test for difference between the two assays yielded a *P*-value of 0.87. If measured CRP changed over calendar time, due to analytical fluctuations, this could bias our risk estimates, but we found no evidence for an interaction between CRP level and date of measurement (= diagnosis). When statistical analyses were run separately by assay, the results were similar; we, therefore, only present combined results in the paper. The assays were assessed daily for precision by using internal controls for each assay (coefficient of variation was 3% at a level of 1.7 mg/L for the nephelometry assay and 6% at a level of 2.1 mg/L for the turbidimetry assay) and monthly for accuracy through a Scandinavian quality control program.

### Statistical analysis

The data were analyzed using STATA statistical software (version 10.1; StataCorp, College Station, TX, USA), and a two-sided *P*-value less than 0.05 was considered statistically significant. On the basis of the measurements of plasma CRP with the nephelometry and turbidemetry assays, we divided patients into assay-specific tertiles of CRP and thereupon combined these assay-specific tertiles into common tertiles of CRP. To test the robustness of our findings, we used the same approach to divide patients into octiles of CRP, and to examine extremely high CRP levels, we divided patients into five groups of CRP levels (0 to 25% percentile, 25 to 50% percentile, 50 to 75% percentile, 75 to 95% percentile, and ≥95% percentile). The decision to examine tertiles as well as robustness using octiles, and extreme phenotypes was decided *a priori*, as we have done previously in similar studies, but for other questions [28,29].

To test whether covariates differed across tertiles of plasma CRP, Kruskal-Wallis one-way analysis of variance was used for continuous covariates and the chi square test was used for categorical covariates. We used the Kaplan-Meier method to plot overall survival, disease-free survival, or cumulative incidence of death from breast cancer and recurrence against follow-up time and used the log-rank test to test for differences across tertiles of CRP. Cox proportional hazards regression models were used to estimate hazard ratios (HRs) and 95% confidence intervals (CIs) of reduced overall and disease-free survival, death from breast cancer, and recurrence as a function of elevated plasma CRP levels. Patients were censored at their date of death (or emigration), dates that are 100% complete in the Danish registries. We used a model that included age at diagnosis only (Model 1), a multifactor-adjusted model that included age at diagnosis, tumour size (≤20 mm, 20 to 50 mm, >50 mm, or unknown), lymph node status (negative, positive, or unknown), presence of distant metastases (yes, no, or unknown), tumour grade (well differentiated, moderately differentiated, poorly/un-differentiated, or unknown), estrogen receptor status (positive, negative, or unknown), progesterone receptor status (positive, negative, or unknown), and HER2 status (negative, positive, or unknown) (Model 2), and a multifactor-adjusted model that included the above-mentioned covariates and the lifestyle factors smoking (<10, 10 to 20, ≥20 cigarettes per day, or unknown), alcohol consumption (≤168 or >168 grams per week, or unknown), and body mass index (<18.5, 18.5 to 24.9, 25 to 29.9, or ≥30.0 kg/m^2^, or unknown) as well as menopause status (pre-menopause, post-menopause, or unknown) and cardiovascular disease (yes, no, or unknown) (Model 3). For trend tests in log-rank and Cox statistics, the CRP groups were coded 1, 2, 3, and so on, corresponding to CRP tertiles and octiles.

For Cox proportional hazards regression analyses, we assessed the assumption of proportional hazards graphically by plotting log(cumulative hazard) as a function of follow-up time. We detected no major violations of the proportional hazards assumption. Interactions between tertiles of plasma CRP and covariates in the regression models was tested for by computing a likelihood ratio test comparing the statistical fit of models with and without a two-factor interaction term.

## Results

Baseline characteristics of the patients by CRP tertiles at the time of diagnosis of breast cancer are shown in Table 1. Levels of CRP were associated with several of these characteristics. The median duration from date of diagnosis to date of blood sampling was seven days (IQR, two to nine days). Table 2 shows the risk of reduced overall and disease-free survival, death from breast cancer, and recurrence by age at diagnosis, prognostic tumour characteristics, lifestyle factors, menopause status, and presence of cardiovascular disease. Causes of deaths in the present cohort were breast cancer (64%), other cancer (11%), cardiovascular disease (11%), respiratory disease (4%), other disease (11%), and unknown cause (1%).

**Table 1:** Baseline characteristics of breast cancer patients by plasma levels of C-reactive protein

	Tertiles of C-reactive protein (mg/L)			
	1st (<1.04)	2nd (1.04 to 3.24)	3rd (>3.24)	*P*
No. of patients	975	966	969	
Median age, years (IQR)	59 (48 to 68)	64 (55 to 73)	65 (56 to 74)	<0.001
Tumour size, No. (%)				
≤20 mm	587 (60)	487 (50)	427 (44)	
20 to 50 mm	297 (30)	377 (39)	384 (40)	
>50 mm	25 (3)	31 (3)	47 (5)	<0.001
Unknown	66 (7)	71 (7)	111 (11)	
Lymph node status, No. (%)				
Lymph node negative	459 (47)	453 (47)	442 (44)	
Lymph node positive	451 (46)	443 (46)	441 (46)	0.74
Unknown	65 (7)	70 (7)	106 (11)	
Distant metastases, No. (%)				
No	942 (97)	929 (96)	909 (94)	
Yes	6 (1)	7 (1)	27 (3)	<0.001
Unknown	27 (3)	30 (3)	33 (3)	
Tumour grade, No. (%)				
Well differentiated	248 (25)	243 (25)	194 (20)	
Moderately differentiated	431 (44)	392 (41)	439 (45)	
Poorly/un-differentiated	158 (16)	193 (20)	154 (16)	0.006
Unknown	138 (14)	138 (14)	182 (19)	
Estrogen receptor status, No. (%)				
Positive	766 (79)	744 (77)	723 (75)	
Negative	146 (15)	150 (16)	140 (14)	0.90
Unknown	63 (6)	72 (7)	106 (11)	
Progesterone receptor status, No. (%)				
Positive	492 (50)	451 (47)	440 (45)	
Negative	243 (25)	286 (30)	258 (27)	0.07
Unknown	240 (25)	229 (24)	271 (28)	
HER2 status, No. (%)				
Positive	279 (29)	260 (27)	268 (28)	
Negative	82 (8)	68 (7)	70 (7)	0.76
Unknown	614 (63)	638 (66)	631 (65)	
Cigarettes smoked per day, No. (%)				
<10	689 (71)	624 (65)	531 (55)	
10 to 20	91 (9)	110 (11)	141 (15)	
≥20	61 (6)	80 (8)	110 (11)	<0.001
Unknown	134 (14)	152 (16)	187 (19)	
Alcohol consumption in grams per week*, No. (%)				
≤168	786 (81)	743 (77)	702 (72)	
>168	66 (7)	82 (8)	86 (9)	0.08
Unknown	123 (13)	141 (15)	181 (19)	
Body mass index in kg/m^2†^, No. (%)				
<18.5	46 (5)	16 (2)	15 (2)	
18.5 to 24.9	554 (57)	367 (38)	255 (26)	
25 to 29.9	133 (14)	241 (25)	242 (25)	
≥30	18 (2)	75 (8)	138 (14)	<0.001
Unknown	224 (23)	267 (28)	319 (33)	
Menopause status, No (%)				
Post menopause	640 (66)	795 (82)	825 (85)	
Pre menopause	325 (33)	159 (16)	130 (13)	<0.001
Unknown	10 (1)	12 (1)	14 (1)	
Cardiovascular disease, No (%)				
No	910 (93)	869 (90)	847 (87)	
Yes	38 (4)	60 (6)	78 (8)	<0.001
Unknown	27 (3)	37 (4)	44 (5)	

Blood samples were drawn and CRP measured at time of diagnosis of breast cancer.

*P*-values were calculated using χ^2 ^tests for categorical variables and Kruskal-Wallis one-way analysis of variance tests for continuous variables. For the evaluation of *P-*values, patients with unknown values were excluded.

IQR = interquartile range.

*Cut-off values are based on the recommendation from of the Danish National Board of Health.

^† ^Body mass index was calculated as weight in kilograms divided by height in meters squared.

**Table 2:** Prognoses after diagnosis of breast cancer by tumour characteristics and lifestyle factors

	Overall survival	Disease-free survival	Death from breast cancer	Recurrence
Prognostic and lifestyle factors	Hazard ratio (95% CI)			
Age, years				
<57	1.00	1.00	1.00	1.00
57 to 69	1.51 (1.12 to 2.03)	1.19 (0.90 to 1.56)	1.13 (0.79 to 1.62)	0.61 (0.40 to 0.93)
≥69	3.20 (2.45 to 4.18)	2.25 (1.76 to 2.87)	2.05 (1.49 to 2.82)	0.56 (0.36 to 0.88)
Tumour size, mm				
≤20	1.00	1.00	1.00	1.00
20 to 50	2.39 (1.89 to 3.01)	2.12 (1.72 to 2.62)	3.27 (2.37 to 4.51)	1.49 (1.03 to 2.17)
>50	3.48 (2.22 to 5.44)	2.44 (1.53 to 3.90)	4.27 (2.33 to 7.82)	0.95 (0.30 to 3.03)
Lymph node status				
Lymph node negative	1.00	1.00	1.00	1.00
Lymph node positive	1.47 (1.18 to 1.84)	1.48 (1.21 to 1.82)	2.49 (1.81 to 3.43)	2.08 (1.41 to 3.07)
Distant metastases				
No	1.00	NA	1.00	NA
Yes	8.57 (5.61 to 13.1)	NA	12.32 (7.66 to 19.80)	NA
Histopathological grade				
Well differentiated	1.00	1.00	1.00	1.00
Moderately differentiated	1.64 (1.19 to 2.26)	1.57 (1.16 to 2.11)	2.52 (1.53 to 4.13)	1.94 (1.05 to 3.59)
Poorly/un-differentiated	2.76 (1.96 to 3.90)	3.00 (2.18 to 4.12)	5.28 (3.17 to 8.79)	5.32 (2.87 to 9.87)
Estrogen receptor status				
Positive	1.00	1.00	1.00	1.00
Negative	2.09 (1.63 to 2.66)	1.98 (1.57 to 2.49)	3.03 (2.24 to 4.10)	2.31 (1.54 to 3.47)
Progesterone receptor status				
Positive	1.00	1.00	1.00	1.00
Negative	2.32 (1.77 to 3.05)	2.02 (1.58 to 2.60)	3.28 (2.25 to 4.77)	1.91 (1.23 to 2.95)
HER2 receptor status				
Positive	1.00	1.00	1.00	1.00
Negative	2.46 (1.38 to 4.39)	2.06 (1.21 to 3.52)	2.72 (1.30 to 5.69)	1.49 (0.58 to 3.85)
No. of cigarettes smoked per day				
<10	1.00	1.00	1.00	1.00
10 to 20	1.30 (0.97 to 1.75)	1.15 (0.86 to 1.54)	1.55 (1.09 to 2.21)	0.96 (0.55 to 1.66)
≥20	1.06 (0.74 to 1.51)	0.96 (0.68 to 1.36)	0.81 (0.49 to 1.37)	0.73 (0.37 to 1.45)
Alcohol consumption in grams per week*				
≤168	1.00	1.00	1.00	1.00
>168	0.79 (0.53 to 1.19)	0.72 (0.48 to 1.08)	0.96 (0.59 to 1.55)	0.83 (0.42 to 1.65)
Body mass index in kg/m^2†^				
<18.5	2.21 (1.37 to 3.57)	2.09 (1.31 to 3.32)	1.10 (0.48 to 2.52)	0.32 (0.04 to 2.29)
18.5 to 24.9	1.00	1.00	1.00	1.00
25 to 29.9	1.06 (0.80 to 1.40)	1.09 (0.84 to 1.42)	1.05 (0.74 to 1.49)	1.16 (0.74 to 1.82)
≥30	1.45 (1.01 to 2.09)	1.58 (1.13 to 2.21)	1.62 (1.05 to 2.52)	1.88 (1.08 to 3.25)
Menopause status, No (%)				
Post menopause	1.00	1.00	1.00	1.00
Pre menopause	0.36 (0.26 to 0.51)	0.56 (0.42 to 0.74)	0.52 (0.36 to 0.77)	1.64 (1.12 to 2.42)
Cardiovascular disease, No (%)				
No	1.00	1.00	1.00	1.00
Yes	2.46 (1.77 to 3.42)	2.32 (1.67 to 3.22)	1.70 (1.03 to 2.79)	1.45 (0.71 to 2.98)

*Cut-off values are based on the recommendation from of the Danish National Board of Health.

^† ^Body mass index was calculated as weight in kilograms divided by height in meters squared.

Hazard ratios are unadjusted.

NA: not applicable.

### Overall survival

**Figure 2:**
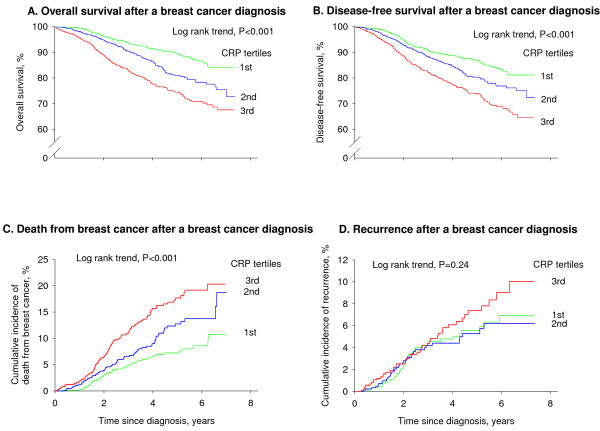
**Overall survival (A), disease-free survival (B), death from breast cancer (C), and cumulative incidence of recurrence after a breast cancer diagnosis (D)**. Results are shown by tertiles of plasma C-reactive protein (CRP) and are based on 2,910 women from the Copenhagen Breast Cancer Study who were observed for up to seven years.

**Figure 3:**
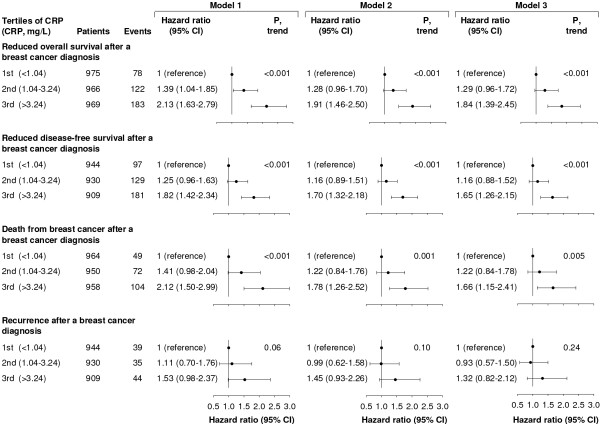
**Risk of reduced overall survival, disease-free survival, and recurrence after a breast cancer diagnosis**. Hazard ratios by tertiles of plasma C-reactive protein (CRP) are based on 2,910 women from the Copenhagen Breast Cancer Study who were observed for up to seven years after a diagnosis of breast cancer. Model 1: adjusted for age at diagnosis. Model 2: adjusted for age at diagnosis, tumour size, lymph node status, presence of distant metastases, tumour grade, and estrogen receptor, progesterone receptor, and HER2 status. Model 3: adjusted for the above-mentioned covariates and smoking, alcohol consumption, body mass index, menopausal status, and cardiovascular disease.

The overall survival among breast cancer patients decreased with increasing levels of CRP (Figure 2A; log-rank trend, *P *< 0.001). The five-year survival rates were 90%, 81%, and 74% among women in the lowest, middle, and highest tertile of CRP. Corresponding age-adjusted HRs were 1.00, 1.39 (95% CI, 1.04 to 1.85), and 2.13 (1.63 to 2.79); *P *for trend <0.001 (Figure 3). Multifactorial adjustment for prognostic tumour characteristics (Model 2) slightly reduced the HRs to 1.00, 1.28 (0.96 to 1.70), and 1.91 (1.46 to 2.50); *P *for trend <0.001. Additional adjustment for lifestyle factors, menopause status, and cardiovascular disease (Model 3) resulted in similar HRs of 1.00, 1.29 (0.96 to 1.72), and 1.84 (1.39 to 2.45); *P *for trend <0.001.

### Disease-free survival

The disease-free survival among breast cancer patients decreased with increasing levels of CRP (Figure 2B; log-rank trend, *P *< 0.001). The five-year disease-free survival rates were 87%, 80%, and 74% among women in the lowest, middle, and highest tertile of CRP. Corresponding age-adjusted HRs were 1.00, 1.25 (95% CI, 0.96 to 1.63), and 1.82 (1.42 to 2.34); *P *for trend < 0.001 (Figure 3). Multifactorial adjustment for prognostic tumour characteristics (Model 2) slightly reduced the HRs to 1.00, 1.16 (0.89 to 1.51), and 1.70 (1.32 to 2.18); *P *for trend <0.001. Additional adjustment for lifestyle factors, menopause status, and cardiovascular disease (Model 3) resulted in similar HRs of 1.00, 1.16 (0.88 to 1.52), and 1.65 (1.26 to 2.15); *P *for trend <0.001.

### Death from breast cancer

The cumulative incidence of death from breast cancer among breast cancer patients increased with increasing levels of CRP (Figure 2C; log-rank trend, *P *< 0.001) reaching 11%, 19%, and 20% among women in the lowest, middle, and highest tertile of CRP at the end of follow-up. Corresponding age adjusted HRs were 1.00, 1.41 (95% CI, 0.98 to 2.04), and 2.12 (1.50 to 2.99); *P *for trend <0.001 (Figure 3). Multifactorial adjustment for prognostic tumour characteristics (Model 2) slightly reduced the HRs to 1.00, 1.22 (0.84 to 1.76), and 1.78 (1.26 to 2.52); *P *for trend = 0.001. Additional adjustment for lifestyle factors, menopause status, and cardiovascular disease (Model 3) resulted in similar HRs of 1.00, 1.22 (0.84 to 1.78), and 1.66 (1.15 to 2.41); *P *for trend = 0.005.

### Recurrence

The cumulative incidence of recurrence among breast cancer patients was highest among women in the highest tertile of CRP, but the incidence of recurrence did not increase stepwise with increasing levels of CRP (Figure 2D; log-rank trend, *P *= 0.24). The cumulative incidence of recurrence at the end of follow-up was 7%, 6%, and 10% among women in the lowest, middle, and highest tertile of CRP. Corresponding age adjusted HRs were 1.00, 1.11 (95% CI, 0.70 to 1.76), and 1.53 (0.98 to 2.37); *P *for trend = 0.06 (Figure 3). The HRs remained similar after multifactor-adjustment for prognostic tumour characteristics and additional adjustment for lifestyle factors, menopause status, and cardiovascular disease.

### Sensitivity analyses

**Figure 4:**
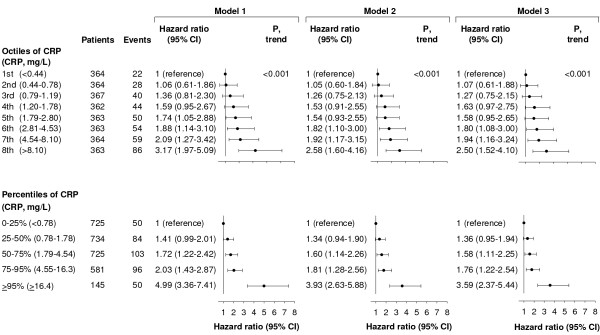
**Risk of reduced overall survival after a breast cancer diagnosis**. Hazard ratios by octiles or extreme levels of plasma C-reactive protein (CRP) are based on 2,910 women from the Copenhagen Breast Cancer Study who were observed for up to seven years after a diagnosis of breast cancer. Model 1: adjusted for age at diagnosis. Model 2: adjusted for age at diagnosis, tumour size, lymph node status, presence of distant metastases, tumour grade, and estrogen receptor, progesterone receptor, and HER2 status. Model 3: adjusted for the above-mentioned covariates and smoking, alcohol consumption, body mass index, menopausal status, and cardiovascular disease.

Dividing CRP levels into octiles also resulted in a stepwise increased risk of reduced overall survival (Figure 4; *P *for trend <0.001). The multifactor-adjusted HR of reduced overall survival among women in the highest versus the lowest octile of CRP was 2.50 (95% CI, 1.52 to 4.10) (Figure 4). Compared to women with CRP levels in the 0 to 25% percentile (<0.78 mg/L), the multifactor-adjusted HR of reduced overall survival was 3.59 (2.37 to 5.44) among women with CRP levels ≥95% percentile (≥16.4 mg/L) (Figure 4).

In sub-analyses stratified for age at diagnosis and prognostic tumour characteristics, elevated CRP levels across tertiles were associated with reduced overall survival irrespective of presence of distant metastases, and estrogen and progesterone receptor status (Table 3). Furthermore, elevated CRP levels were associated with reduced overall survival separately in women above 57 years, with small and middle-sized tumours, positive lymph node status, moderately and poorly/undifferentiated tumours, and HER2 positive tumours. The multifactor-adjusted HR of reduced overall survival for the highest versus the lowest tertile of CRP was 8.63 (2.04 to 36.4) among women with HER2 positive tumours. However, tests of interaction between the CRP tertiles and the prognostic factors were all non-significant, suggesting that elevated CRP levels are associated with similarly reduced overall survival irrespective of age at diagnosis, tumour size, lymph node status, presence of distant metastases, tumour grade, and estrogen receptor, progesterone receptor, and HER2 status (Table 3). Exclusion of patients with CRP values above 40 mg/L, which likely reflect bacterial infection, resulted in similar hazard ratios for all endpoints (data not shown).

**Table 3:** Risk of reduced overall survival by plasma levels of C-reactive protein and prognostic factors

				Tertiles of C-reactive protein (mg/L)			
Stratification	No. of patients	No. of deaths	*P*, inter-action	1st (<1.04)	2nd (1.04 to 3.24)	3rd (>3.24)	*P*, trend
				Multifactor-adjusted hazard ratio (95% CI)			
**None**	2,910	383		1.0	1.29 (0.96 to 1.72)	1.84 (1.39 to 2.45)	<0.001
**Age at diagnosis**							
**<57 years**	964	74	0.40	1.0	0.97 (0.49 to 1.90)	1.69 (0.91 to 3.14)	0.08
**57 to 69 years**	964	103		1.0	1.76 (0.99 to 3.11)	2.53 (1.42 to 4.50)	0.002
**≥69 years**	982	206		1.0	1.18 (0.78 to 1.79)	1.59 (1.07 to 2.37)	0.01
**Tumour size**							
**≤20 mm**	1,501	119	0.33	1.0	1.02 (0.62 to 1.68)	1.66 (1.03 to 2.68)	0.03
**20 to 50 mm**	1,409	264		1.0	1.48 (1.02 to 2.15)	2.04 (1.41 to 2.95)	<0.001
**>50 mm**	103	23		1.0	2.74 (0.36 to 20.7)	0.63 (0.06 to 6.61)	0.44
**Lymph node status**							
**Lymph node negative**	1,334	132	0.59	1.0	1.07 (0.65 to 1.76)	1.48 (0.92 to 2.38)	0.08
**Lymph node positive**	1,335	191		1.0	1.55 (1.01 to 2.38)	2.04 (1.33 to 3.12)	0.001
**Distant metastases**							
**No**	2,780	343	0.66	1.0	1.31 (0.96 to 1.78)	1.80 (1.33 to 2.44)	<0.001
**Yes**	40	23		1.0	0.50 (0.01 to 21.0)	15.6 (1.10 to 221)	0.03
**Tumour grade**							
**Well differentiated**	685	50	0.69	1.0	0.74 (0.31 to 1.75)	1.49 (0.68 to 3.28)	0.21
**Moderately differentiated**	1,262	154		1.0	1.17 (0.74 to 1.88)	1.78 (1.14 to 2.77)	0.007
**Poorly/un-differentiated**	505	92		1.0	2.19 (1.16 to 4.14)	2.23 (1.14 to 4.39)	0.03
**Estrogen receptor status**							
**Positive**	2,233	234	0.83	1.0	1.07 (0.74 to 1.55)	1.59 (1.11 to 2.28)	0.005
**Negative**	436	88		1.0	2.07 (1.08 to 3.98)	2.74 (1.44 to 5.19)	0.002
**Progesterone receptor status**							
**Positive**	1,383	87	0.60	1.0	1.83 (0.93 to 3.59)	2.37 (1.23 to 4.58)	0.01
**Negative**	787	130		1.0	1.47 (0.88 to 2.47)	2.22 (1.33 to 3.71)	0.002
**HER2 status**							
**Positive**	807	29	0.11	1.0	1.64 (0.34 to 7.96)	8.63 (2.04 to 36.4)	<0.001
**Negative**	220	19		1.0	1.24 (0.24 to 6.49)	1.29 (0.31 to 5.45)	0.74

Blood samples were drawn and CRP measured at time of diagnosis of breast cancer.

Hazard ratios are multifactor-adjusted for age at diagnosis, tumour size, lymph node status, presence of distant metastases, tumour grade, and estrogen receptor, progesterone receptor, and HER2 status, smoking, alcohol consumption, body mass index, menopause status, and cardiovascular disease (Model 3), excluding the factor stratified for; however, age stratified analyses were still adjusted for age.

Due to differences in numbers of missing values for the covariates in question, numbers of patients and deaths do not necessarily add up to 2,910 and 383, respectively.

## Discussion

Using a prospective cohort study of 2,910 Danish women with invasive breast cancer, we have demonstrated that elevated CRP levels at the time of diagnosis of breast cancer were associated with reduced overall and disease-free survival and with increased risk of death from breast cancer. These are novel observations.

Mechanistically, three components might explain the observed association between elevated CRP levels and poor breast cancer prognosis. First, tumour cell behavior: plasma CRP levels may reflect the aggressiveness of the tumour, that is, plasma CRP levels might sum up some prognostic information of well-known tumour characteristics, such as tumour stage and grade. In the present study, elevated CRP levels were indeed associated with larger tumour size, presence of distant metastases, and lower tumour grade (although CRP was not linearly associated with tumour grade), and these prognostic factors were associated with poor prognosis. Second, adjacent inflammation: plasma CRP levels might express the magnitude and the nature of any inflammation in the breast tumour microenvironment. Inflammatory pathways play important roles in all stages of tumourigenesis, including tumour initiation and promotion, malignant transformation, tumour invasion, and metastasis [3-6]. Thus, solid tumours typically trigger inflammatory responses that result in the formation of a pro-tumourigenic and pro-angiogenic microenvironment around the tumour. Immune and inflammatory cells in the tumour microenvironment interact with malignant cells in a complicated fashion, the net result of which is stimulation of tumour growth, invasion, and metastasis [5-9]. Despite the fact that breast cancers rarely are characterized by significant histological inflammation, inflammation might also play a role in breast cancer prognosis [15-20]. Thus, macrophage infiltration into invasive breast carcinomas was associated with high vascularity of the breast tumour as well as with reduced recurrence-free and overall survival [18], and targeting of cancer associated fibroblasts resulted in favourable changes of the immune tumour microenvironment and improved anti-metastatic effects of doxorubicin chemotherapy in a murine model of metastatic breast cancer [19]. Furthermore, a recently published study showed that blockade of the IL-8 receptor selectively targets breast cancer stem cells and retards tumour growth and reduces metastasis [17]. Third, host behaviour: plasma CRP levels may outline the general health of the woman at the time of diagnosis of breast cancer. In the present study, elevated CRP levels were indeed associated with smoking and elevated body mass index at the time of diagnosis. However, whereas smoking was not associated with poor breast cancer prognosis, body mass index <18.5 or ≥30 at the time of diagnosis was associated with poor prognosis. Previous studies have shown that elevated CRP levels are associated with all-cause mortality in the general population [29,30]. Thus, it is possible that the present finding of an association between elevated CRP levels at the time of diagnosis of breast cancer and overall survival may not be breast cancer specific but can simply be due to CRP acting as a general marker of health and longevity. However, as 64% of the deaths in the present study were due to breast cancer, breast cancer was the leading cause of death in the present cohort. The fact that the observed association between elevated CRP levels and increased risk of death from breast cancer was of the same magnitude as the observed association between elevated CRP levels and overall survival suggest that CRP may indeed be associated with mortality through a breast-cancer-related mechanism. Elevated plasma CRP is a well-known marker of cardiovascular disease [31], which could imply that elevated CRP levels are associated with overall survival through the association with cardiovascular disease. However, the fact that only 11% of the deaths in the present cohort were due to cardiovascular diseases and the fact that we adjusted for cardiovascular disease at baseline limit the possibility that our finding on overall survival is severely confounded by deaths from cardiovascular disease.

Our findings of a positive association between elevated CRP levels and poor breast cancer prognosis are in contrast to negative studies with 110 to 300 patients [25,26], but are indirectly supported by findings from other studies with 85 to 734 patients which did report a positive association [21-24]. The largest study so far which comprised approximately 700 women treated successfully for early stage breast cancer found that elevated levels of CRP measured two and a half years after the time of diagnosis were associated with reduced disease-free and overall survival [23]. Thus, the combined evidence from this and our own study suggests that elevated plasma CRP levels are associated with short-term as well as long-term prognosis after breast cancer. Whereas studies that measure CRP after diagnosis and treatment reflect long-term prognosis, studies that measure CRP at the time of diagnosis more likely reflect breast cancer specific survival. In fact, approximately 55% of the deaths, 60% of the deaths from breast cancer, and 68% of the recurrences in the present study occurred during the first two and a half years after a diagnosis of breast cancer. Therefore, our results may not be comparable to studies that have measured CRP after diagnosis and treatment.

The large sample size of our study allowed us to stratify for well-established prognostic tumour characteristics, and interestingly our data suggest that elevated CRP levels are associated with reduced overall survival irrespective of age at diagnosis, tumour size, lymph node status, presence of distant metastases, tumour grade, and estrogen receptor, progesterone receptor, and HER2 status. However, we observed a very strong association between elevated CRP levels and reduced overall survival among women with positive HER2 status which may warrant further investigation by future studies.

Dividing plasma CRP levels into octiles resulted in a stepwise increased risk of reduced overall survival, demonstrating the robustness of the observed association between elevated CRP levels and risk of reduced overall survival. Furthermore, we observed that compared to women with CRP levels in the 0 to 25% percentile (CRP <0.78 mg/L), women with CRP levels ≥95% percentile (≥16.4 mg/L) had a 3.5-fold increased risk of reduced overall survival, suggesting that women with high CRP levels at the time of diagnosis have a particularly poor survival.

Although the age-adjusted HR for recurrence for the highest versus the lowest tertile of CRP was borderline significant, we did not detect a statistically significant association between elevated CRP levels and recurrence of breast cancer, which may suggest that no such association exists. However, as we only detected 118 recurrences in our study in contrast to 383 deaths, it is also possible that our statistical power was too small to detect an association. Therefore, other, larger studies are needed to answer unequivocally whether increased CRP levels at the time of diagnosis associate with recurrence of breast cancer or not.

As CRP is one of several acute-phase proteins, whose concentrations increase during acute or chronic inflammation, other inflammation-related biomarkers may also be associated with breast cancer prognosis. Prior studies have reported that lower serum albumin concentrations [25], elevated serum amyloid A concentrations [23], and elevated YKL-40 concentrations [32] are all associated with poor breast cancer prognosis.

So far, the present study is by far the largest study that has examined whether an association exists between CRP levels at the time of diagnosis and breast cancer prognosis. However, certain limitations must be acknowledged. Although we did adjust for potential confounders, we cannot exclude residual confounding by factors not taken into account, such as socioeconomic status, physical activity, and treatment, or by imperfectly or incompletely measured confounders. Since patients are referred to different treatment regimes on the basis of tumour characteristics, which we did adjust for, it seems unlikely that our results are confounded by differences in treatment. Also, we did not measure serum amyloid A, which has previously been associated with an even stronger increase in risk of death than CRP [23]. Finally, since the present study is a single-center study of Danish women diagnosed with breast cancer, our findings may not necessarily apply to other ethnic groups. Since our department for breast surgery treats all patients with breast cancer from a geographically well-defined area of Copenhagen regardless of severity or sub-diagnosis, our results may indeed be generalized to comparable populations of unselected patients with breast cancer.

## Conclusions

In conclusion, in 2,910 Danish women with invasive breast cancer, elevated CRP levels at the time of diagnosis of breast cancer were associated with reduced overall and disease-free survival and with increased risk of death from breast cancer, independently of well-established prognostic tumour characteristics and lifestyle factors.

## Abbreviations

CI: confidence interval; CRP: C-reactive protein; HR: hazard ratio; ICD: International Classification of Diseases; WHO: World Health Organization

## Competing interests

The authors declare that they have no competing interests.

## Authors' contributions

All authors contributed to the conception and design of the study. HF and SEB contributed to the acquisition of data. KHA performed the statistical analysis and drafted the manuscript. BGN, HF and SEB critically revised the manuscript. All authors read and approved the final manuscript.
